# Supplementary figures and images for: Comparison of interventions for Barrett’s esophagus: A network meta-analysis
Source: PLoS One. 2024 May 6;19(5):e0302204. doi: 10.1371/journal.pone.0302204 (PMC11073690; doi:10.1371/journal.pone.0302204)

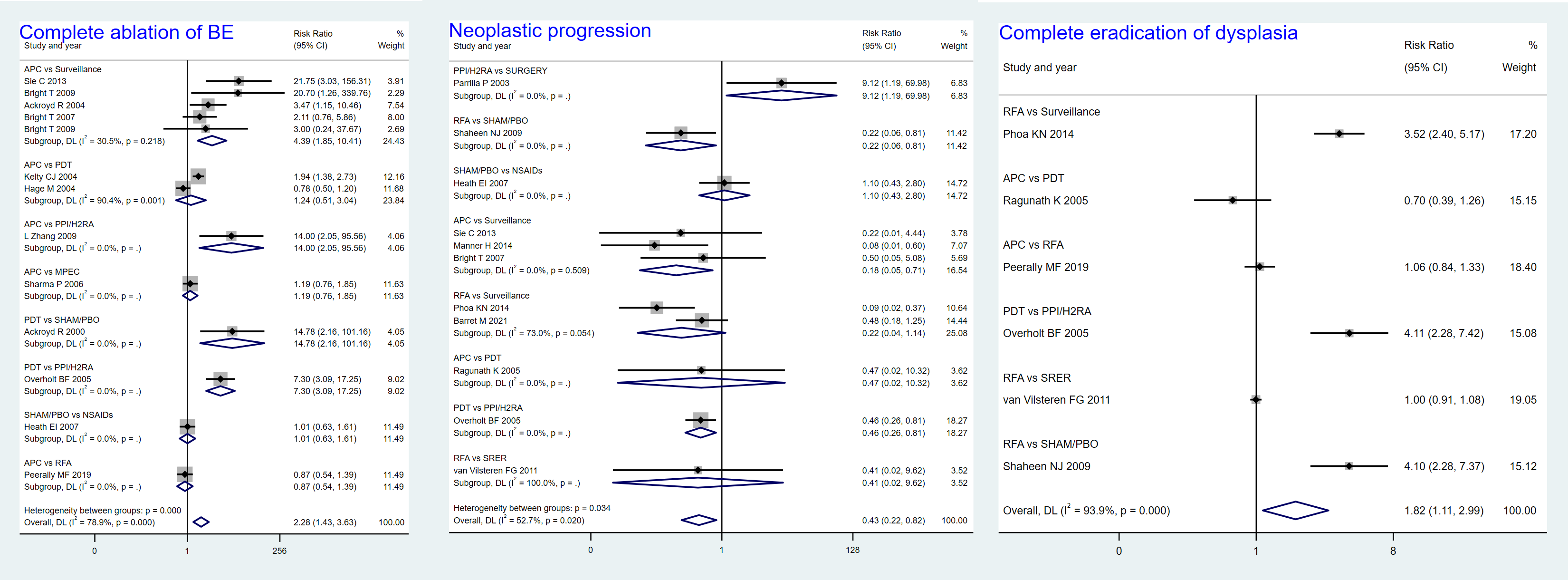

Supplement: S1 Fig — (TIF) [file pone.0302204.s002.tif]

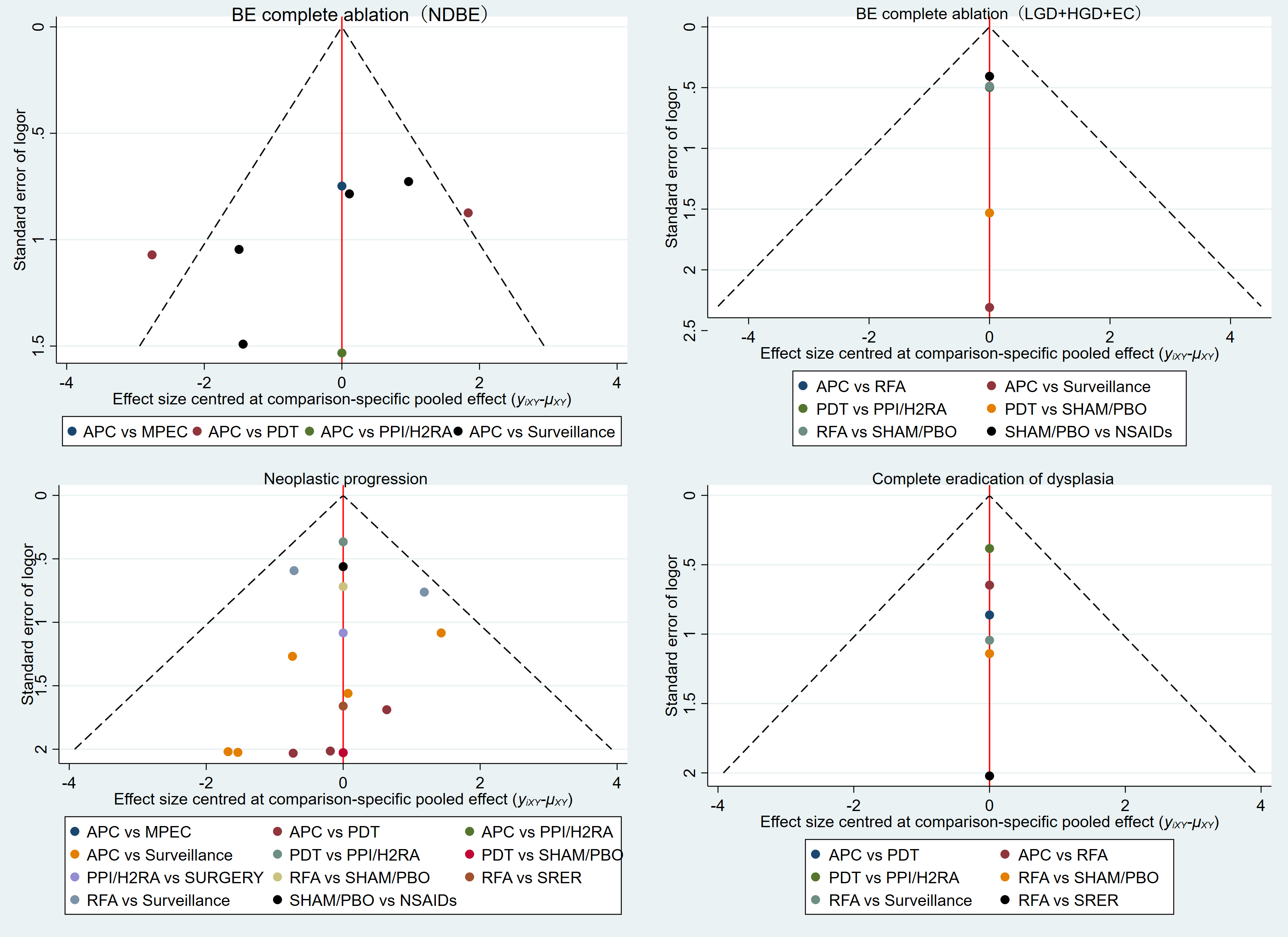

Supplement: S2 Fig — (TIF) [file pone.0302204.s003.tif]
