# Supplementary material for: Comparison of interventions for Barrett’s esophagus: A network meta-analysis
Source: PLoS One. 2024 May 6;19(5):e0302204. doi: 10.1371/journal.pone.0302204 (PMC11073690; doi:10.1371/journal.pone.0302204)
Supplement: Attachment — Submitted filename: Response to Reviewers.docx [file pone.0302204.s005.docx]

Dear Editors and Reviewers:

Thank you for your letter and for the reviewers’ comments concerning our manuscript entitled “Comparison of interventions for Barrett's esophagus: a network meta-analysis” (ID: PONE-D-23-16418). Those comments are all valuable and very helpful for revising and improving our paper, as well as the important guiding significance to our researches. We have studied comments carefully and have made correction which we hope meet with approval.

SUGGESTIONS FROM EDITOR（***1. Please ensure that your manuscript meets PLOS ONE's style requirements, including those for file naming. The PLOS ONE style templates can be found at***

***https://journals.plos.org/plosone/s/file?id=wjVg/PLOSOne_formatting_sample_main_body.pdf and***

***https://journals.plos.org/plosone/s/file?id=ba62/PLOSOne_formatting_sample_title_authors_affiliations.pdf.***

***2. We noticed you have some minor occurrence of overlapping text with the following previous publication(s), which needs to be addressed:***

***https://www.dovepress.com/front_end/getfile.php?fileID=26200***

***https://onlinelibrary.wiley.com/doi/10.1111/j.1365-2036.2004.02277.x***

***In your revision ensure you cite all your sources (including your own works), and quote or rephrase any duplicated text outside the methods section. Further consideration is dependent on these concerns being addressed.***

***3. Please include your tables as part of your main manuscript and remove the individual files. Please note that supplementary tables (should remain/ be uploaded) as separate "supporting information" files.***

***4. Thank you for stating the following financial disclosure:***

***“This is an unfunded study”***

***At this time, please address the following queries:***

***a)	Please clarify the sources of funding (financial or material support) for your study. List the grants or organizations that supported your study, including funding received from your institution.***

***b)	State what role the funders took in the study. If the funders had no role in your study, please state: “The funders had no role in study design, data collection and analysis, decision to publish, or preparation of the manuscript.”***

***c)	If any authors received a salary from any of your funders, please state which authors and which funders.***

***d)	If you did not receive any funding for this study, please state: “The authors received no specific funding for this work.”***

***Please include your amended statements within your cover letter; we will change the online submission form on your behalf.***

***5. Please include captions for your Supporting Information files at the end of your manuscript, and update any in-text citations to match accordingly. Please see our Supporting Information guidelines for more information: http://journals.plos.org/plosone/s/supporting-information.*** ）

Our response to the general comments from the editor：

1. The manuscript has been revised in accordance with PLOS ONE's style requirements.

2. The minor issue of overlapping text with the previously mentioned publications has been resolved.

3. We have integrated the tables into the manuscript and removed the individual files.

4. The authors did not receive any specific funding for this work. We have included our amended statements within cover letter.

5. We have added captions for the Supporting Information files at the end of our manuscript and adjusted any in-text citations accordingly.

Revised portion are marked in red in the paper. The main corrections in the paper and the responds to the reviewer’s comments are as flowing:

**Reviewer #1:**

1. Non-dysplastic BE does not need eradication treatment based on cost-effects ratio. The authors suggest a different opinion refering to referance 37, however, the article can not be searched. A article written by Prateek Sharma,et al. , seems the article the authors referred, according to the observation from Prateek Sharma, there was only 18 patients of 1376 objectives had at least 2 initial consecutive endoscopies with biopsies revealing non-dysplastc mucosa. And the inaccuracy operation of endoscopic biopsy making an impact on the result should be considered, then, this is not strong evident supporting authors opinion. It is better for the authors to review more publishes correctly.

**The author’s answer:** We are truly grateful for your professional review of our article. As per your request, Reference 37 can be accessed at the following address: <https://www.researchgate.net/publication/246664693_Progression_of_Barrett's_esophagus_to_high_grade_dysplasia_and_cancer_preliminary_results_of_the_BEST_Barrett's_Esophagus_Study_Trial> Although the potential impact of inaccuracies in endoscopic biopsy operations on the results should be considered, it is undeniable that this extensive study suggests a need for caution regarding the rapid progression from intestinal metaplasia to invasive carcinoma.

2. Endoscopic surveillace is a effective method to detect early carcinoma also as a uesful management for patients who accepted endoscopic eradication therapy or other treatment. But, It should not be compared with other complete eradication treatment.

**The author’s answer:** Thank you once more for your encouraging feedback and insightful suggestions on enhancing the quality of our manuscript. Regarding patients with non-dysplastic Barrett's esophagus, options such as endoscopic eradication or alternative treatments, along with regular endoscopic surveillance without additional intervention, are considered as potential treatment alternatives. Our aim in comparing these treatment options is to identify the most beneficial approach for the patients.

3. For now, The best treatment for BE should based on the grade of dysplasia and the widths and depths of the lesion. For example, Endoscopic mucosal resection (EMR) is a widely used technique for diagnosing and eradicating superficial BE dysplasia and neoplasia, Radiofrequency ablation (RFA) can be employed to eradicate circumferential areas of dysplastic BE.

**The author’s answer:** I wholeheartedly agree with your opinion. However, as evidenced by the studies presented here, diverse treatment approaches are being employed for the management of patients with BE, perhaps indicative of the absence of a clear consensus. We aim to elucidate the relative priorities of these treatment options through rigorous scientific statistical methods.

4. Photodynamic Therapy is usually applied to esophageal cancer treatment. For BE, it is a option, but not commonly accepted.

**The author’s answer:** Thank you for your feedback. We aim to use our research to identify and advocate for treatments that are more effective than others and promote their widespread use.

5. The authors research seems a little contributions for BE treatment reseach. Obsolete publishes included might be a reason.

**The author’s answer:** We appreciate your constructive feedback. We will make the required revisions to our manuscript to ensure the credibility of our findings.

**Reviewer #2:**

The author analyzed the advantages and disadvantages of different treatment methods for Barrett's esophagus using a net META method. The literature included in the study was all RCT studies, with small differences in sample size and high reliability in each study. Preliminary analysis has been conducted on the advantages of current methods for treating Barrett's esophagus. However, there are still some shortcomings in this article. In the discussion section, the current Barrett's esophageal treatment guidelines were not combined to point out the similarities and differences between your research and the guidelines.

**The author’s answer:** On behalf of all the contributing authors, I would like to express our sincere appreciation for your constructive comments regarding our article. Your comments have been immensely valuable and have greatly aided in enhancing the quality of our work. In response to your feedback, we have made substantial modifications to our manuscript to bolster the credibility of our results. In this revised version, all changes have been clearly highlighted in red within the document. We are truly grateful for your positive feedback and invaluable suggestions, which have undoubtedly contributed to the overall improvement of our manuscript.

**Reviewer #3:**

1. Please insert a space before the brackets when introducing an abbreviation.

**The author’s answer:** We would like to express our gratitude for the time you've invested and for granting us the opportunity to enhance the manuscript. We have addressed the formatting issues in the article.

2. In the methods section "study selection", please define what you consider as a small sample size.

**The author’s answer:** We define small sample size studies as those with less than 20.

3. Statistical analysis: Here you write that it was investigated to what extent a small-sized trial influenced the results. However, in the study selection section small sized trials were excluded, please explain the discrepancy between these two points.

**The author’s answer:** To ensure the quality of the included Randomized Controlled Trials (RCTs), we excluded studies with small sample sizes below 20. Additionally, we constructed comparison-adjusted funnel plots and checked for symmetry to evaluate whether studies with smaller sample sizes among those included in the article influenced efficacy results.

4. Table 1: It would be nice, if you could explain in the legend, what is meant by wrong outcome, as this term is slightly misleading.

**The author’s answer:** Due to the variety of techniques and treatment regimens employed for Barrett's esophagus, we categorized all treatments into 10 major groups based on the selected studies, as detailed in Table 1.

5. MPEC is not introduced in the text.

**The author’s answer:** Thank you for your suggestion. We have included an introduction to MPEC in the article and highlighted it in red.

6. For figure 2: Please consider indicating, which of the 23 studies you have considered as potentially biased.

**The author’s answer:** Based on your valuable suggestions, we have incorporated six studies with a high risk of bias into the quality assessment section of the article and highlighted them using red font. Studies with four or more high-risk domains were deemed to possess an overall high risk of bias.

7. It is slightly irritating, that Fig. 4A and 5A are mentioned way earlier in the text then the following numbers.

**The author’s answer:** Thank you for your comments, which are greatly appreciated. I'd like to clarify that in our effort to systematically compare the effectiveness of various treatment options, we have established four different outcome indicators. To enhance readability for our readers, we have standardized the serial numbers of the associated pictures for each outcome indicator.

8. Figure legend 2 needs a little more information what is to see in the figure.

**The author’s answer:** Thank you for your comment, which we have supplemented in the manuscript.

9. Reference in text to supplement figure 2 is missing.

**The author’s answer:** We sincerely thank you for your valuable feedback that we have used to improve the quality of our manuscript.

10. Also discuss newer developments like Hybrid-APC:

Shimizu, T., Samarasena, J. B., Fortinsky, K. J., Hashimoto, R., Chehade, N. E. H., Chin, M. A., ... & Chang, K. J. (2021). Benefit, tolerance, and safety of hybrid argon plasma coagulation for treatment of Barrett's esophagus: US pilot study. Endoscopy International Open, 9(12), E1870-E1876.

Knabe, M., Beyna, T., Rösch, T., Bergman, J., Manner, H., May, A., ... & Ehlken, H. (2022). Hybrid APC in Combination With Resection for the Endoscopic Treatment of Neoplastic Barrett's Esophagus: A Prospective, Multicenter Study. The American journal of gastroenterology, 117(1), 110-119.

**The author’s answer:** Limited attention had been given to Hybrid-APC. The research progress of Hybrid-APC has now been incorporated into the discussion section of the article.

11. In your overall conclusion I am missing a statement regarding surgery as a therapeutic option.

**The author’s answer:** Thank you for your suggestion. Given that only one of the studies we analyzed involved surgery as an intervention, we approached making comparisons between surgery and other treatment options in the Discussion section with caution. It is undeniable that endoscopic treatments maintain the normal esophageal anatomy, are safer, and are linked to lower morbidity and mortality rates compared to esophagectomy. For these reasons, endoscopic interventions are increasingly becoming the primary therapy for patients with high-grade dysplasia (HGD) or intramucosal cancer, replacing surgery.

**Reviewer #4:**

1. In the "study selection", the article divided the treatments into 10 major groups. I wonder if it could have been possible to group similar treatments together to create fewer groups.

**The author’s answer:** Thank you for your valuable feedback, which is greatly appreciated. Prior to this, we had made attempts to categorize similar interventions, such as grouping proton pump inhibitors and H2 receptor antagonists together. Further consolidation could potentially impact the accuracy of the research findings.

2. In the "study selection", it was mentioned that studies with small sample sizes were excluded. It would be helpful to specify the criteria used to determine what is considered a small sample size.

**The author’s answer:** Thanks for your suggestion, we define small sample size studies as those with less than 20. We have added it in the article and marked it in red.

3. While there seems to be a fundamental difference in the meaning of "complete ablation of BE" and "complete eradication of dysplasia," in practical terms, they appear to have very similar criteria. It would be beneficial to mention the rationale for separating these two, as they seem quite similar in clinical application.

**The author’s answer:** Thanks for your suggestion, we have added it to Study Selection of the article and marked it in red.

4. In Figure 1, the number of cases classified as "irrelevant" is quite high (805 cases). It would be helpful if the specific criteria used to make this determination were explicitly stated.

**The author’s answer:** Thank you for your comment. We define irrelevant studies as those that are not related to the treatment of Barrett's esophagus.

5. Generally, dysplasia and esophageal adenocarcinoma are assessed using different criteria. If the article refers to "complete ablation of BE" and places adenocarcinoma on the same level as dysplasia, it would be useful to explain the reasoning behind this.

**The author’s answer:** When it comes to the complete ablation of Barrett's esophagus, we combined adenocarcinoma and dysplasia into a single group for statistical analysis, given the limited number of studies and the small sample size specifically related to adenocarcinoma.

6. If the article suggests that adenocarcinoma can progress directly from metaplasia without going through dysplasia, it might be more helpful to mention this in the introduction or study design section rather than just in the discussion. This could help reduce confusion.

**The author’s answer:** We would like to express our gratitude for your time and the valuable opportunity you have provided us to enhance the manuscript. Our study has indicated that depending solely on endoscopic surveillance or PPI/H2RA treatment alone might not be sufficient for managing BE. This conclusion is supported by the research of SHARMA P et al., who noted that a subset of patients with intestinal metaplasia could progress rapidly to invasive carcinoma without the usual progression through dysplasia. These insights have been integrated into the discussion section of the article.
